# The role of COPD in survival of NSCLC patients receiving immune checkpoint inhibitors: A meta-analysis

**DOI:** 10.17305/bb.2025.12355

**Published:** 2025-05-26

**Authors:** Yan Zhu, Chongyang Wang

**Affiliations:** 1 Department of Emergency Medicine, Shaoxing Seventh People’s Hospital, Shaoxing, China

**Keywords:** Non-small cell lung cancer, NSCLC, chronic obstructive pulmonary disease, COPD, immune checkpoint inhibitors, ICIs, survival, meta-analysis

## Abstract

The impact of chronic obstructive pulmonary disease (COPD) on the survival of patients with non-small cell lung cancer (NSCLC) receiving immune checkpoint inhibitors (ICIs) remains unclear. Given the growing use of ICIs in NSCLC treatment and the high prevalence of COPD among these patients, understanding this relationship is essential. This meta-analysis aims to evaluate the association between COPD and survival outcomes in NSCLC patients treated with ICIs. A systematic search was conducted in PubMed, Embase, and Web of Science from inception to February 10, 2025. Observational studies reporting survival outcomes in NSCLC patients with and without COPD undergoing ICI therapy were included. Hazard ratios (HRs) with 95% confidence intervals (CIs) were pooled using a random-effects model to account for heterogeneity. Thirteen retrospective cohort studies involving 5564 patients were included. COPD was associated with improved progression-free survival (PFS) (HR: 0.68, 95% CI: 0.54–0.85, *P* < 0.001) and overall survival (OS) (HR: 0.80, 95% CI: 0.68–0.95, *P* ═ 0.01) in NSCLC patients receiving ICIs. Heterogeneity was moderate (*I*^2^ ═ 46% for PFS, *I*^2^ ═ 43% for OS). Subgroup analyses indicated that the association between COPD and survival outcomes was consistent across study regions (Asian vs Western countries), patient age, sex distribution, COPD diagnostic criteria (spirometry, clinical diagnosis, or CT-diagnosed emphysema), follow-up duration, analytic models (univariate vs multivariate), and study quality scores (*P* for subgroup differences >0.05). Furthermore, univariate meta-regression analysis showed no significant modification of results by sample size, mean age, sex distribution, follow-up duration, or study quality scores (all *P* > 0.05).

## Introduction

Lung cancer remains the leading cause of cancer-related mortality worldwide, with non-small cell lung cancer (NSCLC) accounting for approximately 85% of all cases [1, 2]. Despite advancements in diagnostic techniques and treatment modalities, the prognosis for NSCLC remains poor, particularly among patients with advanced-stage disease [3]. Historically, chemotherapy and targeted therapies have been the mainstays of treatment, but in recent years, immune checkpoint inhibitors (ICIs) have transformed the therapeutic landscape [4, 5]. By targeting programmed cell death protein 1 (PD-1), programmed death-ligand 1 (PD-L1), or cytotoxic T-lymphocyte-associated protein 4 (CTLA-4), ICIs restore antitumor immune responses [6], leading to significant survival benefits in selected NSCLC patients [7]. However, responses to ICIs vary widely, underscoring the need to identify predictive factors that can guide patient selection and improve therapeutic outcomes [8]. Chronic obstructive pulmonary disease (COPD), a progressive inflammatory lung condition marked by persistent airflow limitation, is highly prevalent in NSCLC populations, with reported rates ranging from 40% to 70% [9, 10]. Shared risk factors—particularly cigarette smoking—contribute to this strong association [11]. Traditionally, COPD has been considered a negative prognostic factor in NSCLC due to its association with increased perioperative complications, higher risks of pulmonary infections, and reduced tolerance to chemotherapy and radiotherapy [12]. Several observational studies [13, 14] and a meta-analysis 15 have linked COPD with poorer survival outcomes in NSCLC. However, most of this research has focused on patients receiving conventional treatments, while those undergoing ICI therapy have been largely under-represented [15]. This knowledge gap has led to ongoing uncertainty about the effect of COPD on ICI efficacy in NSCLC. Emerging evidence suggests that COPD may not uniformly exert a negative impact on NSCLC outcomes, particularly in the context of ICI therapy [16]. Recent studies indicate that COPD-related immune dysregulation might actually enhance the effectiveness of ICIs in lung cancer [17]. COPD is associated with chronic inflammation and modifications in the tumor microenvironment, which may influence immune responses [18]. Specifically, patients with COPD often exhibit increased PD-L1 expression on tumor cells and elevated tumor mutational burden (TMB), both established biomarkers of ICI responsiveness [17, 19]. Additionally, COPD has been linked to shifts in immune cell composition, including greater CD8+ T cell infiltration and heightened immune activation, which could enhance the antitumor effects of ICIs [17, 19]. Some clinical studies have reported improved survival outcomes in NSCLC patients with coexisting COPD treated with ICIs [20–29], though not all findings have reached statistical significance. These observations challenge the long-standing view that COPD universally worsens lung cancer prognosis. However, the current evidence remains inconsistent [30–32], and no comprehensive meta-analysis has systematically evaluated the impact of COPD on ICI efficacy in NSCLC. To address this gap, the present meta-analysis aims to assess the influence of COPD on survival outcomes—specifically progression-free survival (PFS) and overall survival (OS)—in NSCLC patients receiving ICIs.

## Materials and methods

The study adhered to the PRISMA 2020 guidelines [33, 34] and the Cochrane Handbook for Systematic Reviews and Meta-Analyses [35] in conducting this meta-analysis. This included the study protocol design, data extraction, statistical analysis, and results presentation. The meta-analysis protocol was registered with the International Prospective Register of Systematic Reviews (PROSPERO) under the identifier CRD420251006457.

### Literature search

To identify studies relevant to this meta-analysis, we conducted a comprehensive literature search of PubMed, Embase, and Web of Science from database inception to February 10, 2025. The search strategy incorporated a broad range of terms related to the exposure, disease condition, and intervention of interest. Specifically, the following keywords and their synonyms were used: (1) chronic obstructive pulmonary disease OR “COPD” OR “chronic obstructive lung disease” OR “chronic obstructive airway disease” OR “emphysema” OR “chronic airflow limitation” OR “chronic airway obstruction”; (2) non-small cell lung cancer OR “NSCLC” OR “non-small cell carcinoma” OR “lung adenocarcinoma” OR “lung squamous cell carcinoma” OR “lung neoplasms”; and (3) immune checkpoint inhibitors OR “ICI” OR “programmed cell death protein 1” OR “PD-1” OR “PD-L1” OR “cytotoxic T-lymphocyte-associated protein 4” OR “CTLA-4” OR the names of individual agents, including “pembrolizumab,” “nivolumab,” “atezolizumab,” “durvalumab,” “cemiplimab,” “avelumab,” “ipilimumab,” and “tremelimumab.” The search was limited to studies involving human subjects, published in English, and appearing as full-length articles in peer-reviewed journals. Additionally, the reference lists of included original and review articles were manually screened to identify further eligible studies. The complete search strategies for each database are provided in Supplemental data.

### Inclusion and exclusion criteria

The inclusion criteria for potential studies were defined according to the PICOS framework:

P (Patients): Adult patients (aged 18 years or older) with confirmed diagnosis of NSCLC receiving ICIs, regardless of the cancer histology, stage, or previous anticancer treatments.

I (Exposure): Patients with COPD. The methods for the validation of COPD were consistent with those used in the original studies.

C (Comparison): Patients without COPD.

O (Outcome): Survival outcomes, including OS and PFS, compared between patients with and without COPD. In general, PFS is defined as the time from ICIs treatment initiation to disease progression or death, whichever occurs first, while OS is defined as the time from ICIs treatment initiation to death from any cause.

S (Study design): Observational studies with longitudinal follow-up, such as cohort studies, nested case-control studies, or post-hoc analyses of clinical trials.

Studies were excluded if they were reviews, editorials, meta-analyses, or preclinical research, or if they did not exclusively include patients with NSCLC, did not involve treatment with ICIs, lacked COPD as an exposure, or did not report the relevant survival outcomes. For studies with overlapping populations, the one with the largest sample size was included in the meta-analysis.

### Study quality assessment and data extraction

The literature search, study selection, quality assessment, and data extraction were independently conducted by two authors. Discrepancies were resolved through discussion and consensus. Study quality was assessed using the Newcastle-Ottawa Scale (NOS) [36], which evaluates selection, control of confounding factors, and outcome measurement and analysis. Scores range from 1 to 9, with a score of nine indicating the highest quality. Studies with NOS scores of seven or higher were generally considered high quality [36]. Extracted data included study characteristics (author, year, country, and design), participant details (number of patients, mean age, sex, cancer stage, and ICIs used), methods for COPD diagnosis and the number of patients with COPD in each study, median follow-up durations, and variables adjusted for when evaluating the association between COPD and survival outcomes in patients with NSCLC receiving ICIs.

**Figure 1.**
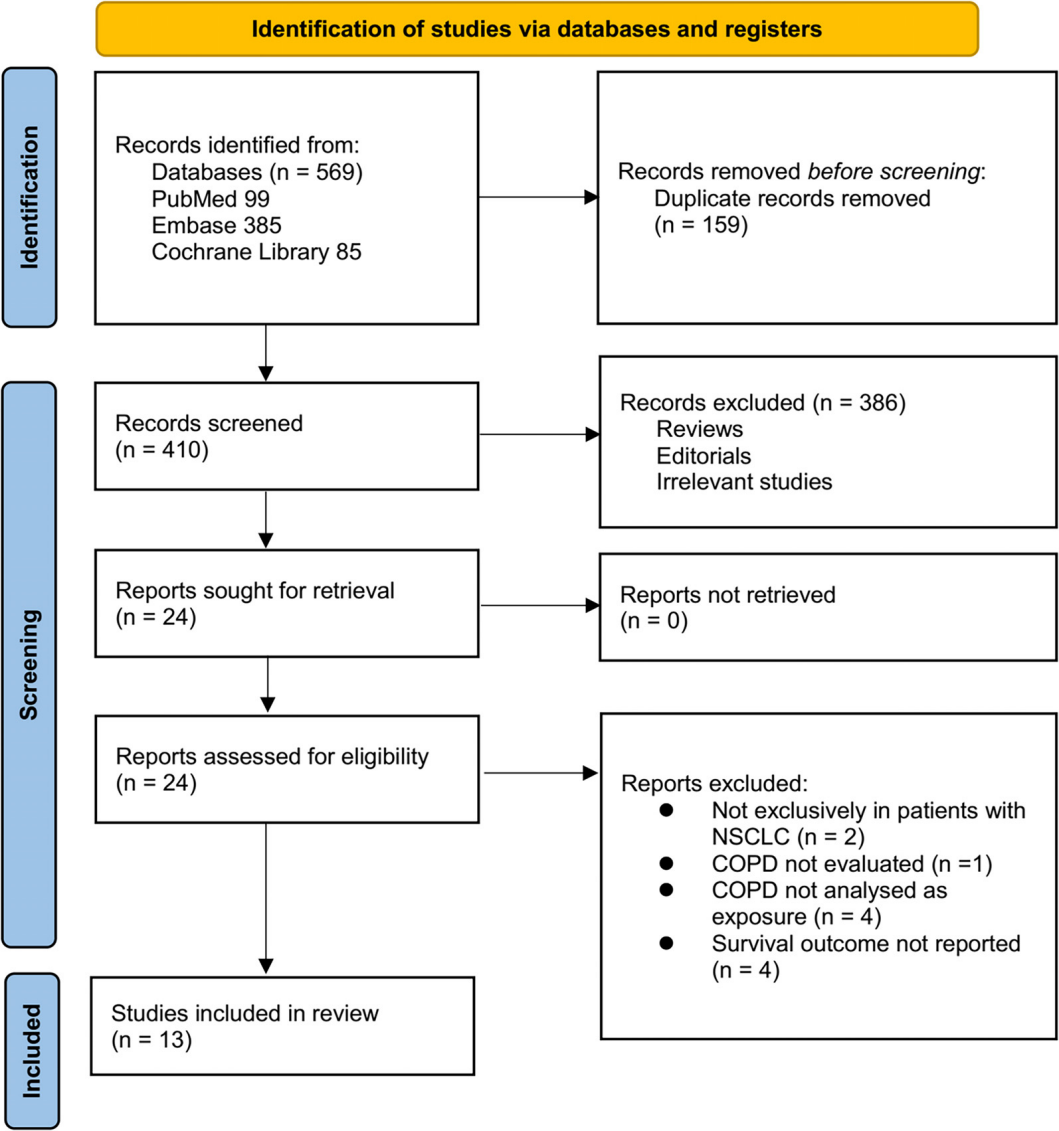
**Flowchart of database search and study inclusion**. NSCLC: Non-small cell lung cancer; COPD: Chronic obstructive pulmonary disease.

### Statistical analysis

The associations between COPD and PFS or OS in patients with NSCLC receiving ICIs were summarized using hazard ratios (HRs) and corresponding 95% confidence intervals (CIs), comparing patients with and without COPD. HRs and their standard errors were derived from 95% CIs or *P* values and subsequently log-transformed to stabilize variance and normalize the distribution [35]. Heterogeneity was assessed using the Cochrane *Q* test and *I*^2^ statistic [37], with *I*^2^ values of <25%, 25%–75%, and >75% indicating low, moderate, and high heterogeneity, respectively. A random-effects model was applied to account for between-study variability [35]. Sensitivity analyses were conducted by sequentially excluding individual studies to assess the robustness of the findings. Subgroup analyses were also performed to examine the impact of study characteristics on outcomes, including study region (Asian vs Western countries), mean patient age, proportion of male participants, COPD diagnostic methods, median follow-up duration, type of analytic model (univariate vs multivariate), and NOS scores. For continuous variables, medians were used as cutoff points to define subgroups. Additionally, univariate meta-regression analyses were conducted to evaluate whether the association between COPD and survival outcomes was significantly modified by variables, such as sample size, mean age, proportion of men, follow-up duration, and NOS scores [35]. Publication bias was assessed using funnel plots and visual inspection for asymmetry, supplemented by Egger’s regression test [38]. All analyses were conducted using RevMan (Version 5.1; Cochrane Collaboration, Oxford, UK) and Stata software (Version 12.0; Stata Corporation, College Station, TX, USA).

**Table 1:** Characteristics of the included cohort studies

**Study**	**Country**	**Design**	**No. of patients included**	**Mean age (years)**	**Men (%)**	**Cancer stage**	**ICIs used**	**Methods for the diagnosis of COPD**	**No. of patients with COPD**	**Median follow-up duration (months)**	**Outcomes reported**	**Variables adjusted**
Biton, 2018	France	RC	39	68.7	61.5	IIIB–IV	Nivolumab	Postbronchodilator FEV1/FVC ratio <0.7	19	21	PFS and OS	None
Mark, 2018	USA	RC	125	66.4	56.8	III–IV	Nivolumab, pembrolizumab, atezolizumab, or avelumab	Clinically diagnosed COPD	60	20	PFS and OS	Age, sex, smoking status, previous history of CAD, previous anticancer treatment, and BMI
Shin, 2019	Korea	RC	133	62.9	75.9	IV	Pembrolizumab	Postbronchodilator FEV1/FVC ratio <0.7	59	12	PFS and OS	Age, sex, smoking, ECOG PS, tumor histology, and previous anticancer treatment
Isono, 2020	Japan	RC	180	68.5	77.8	III–IV	Nivolumab, pembrolizumab, or atezolizumab	CT diagnosed pulmonary emphysema	99	10	OS	None
Takayama, 2021	Japan	RC	153	68	75.2	III to IV	Nivolumab, pembrolizumab, or atezolizumab	CT diagnosed pulmonary emphysema	71	20	PFS and OS	Age, sex, smoking, ECOG PS, tumor histology, previous anticancer treatment, and PD-L1 expression status
Zeng, 2022	China	RC	122	66	95.1	IV	Nivolumab, or pembrolizumab	Clinically diagnosed COPD	61	20	PFS and OS	None
Noda, 2022	Japan	RC	56	70.8	35.7	IIIB–IV	Nivolumab, pembrolizumab, or atezolizumab	CT diagnosed pulmonary emphysema	41	20	PFS and OS	Age, sex, smoking, ECOG PS, tumor histology, previous anticancer treatment, and PD-L1 expression status
Zhang, 2022	China	RC	99	64.9	92.9	IIIB–IV	NR	Postbronchodilator FEV1/FVC ratio <0.7	80	42	PFS	None
Dong, 2024	China	RC	74	63.4	87.8	I–IIIB	Nivolumab, pembrolizumab, atezolizumab, tirellizumab, toripalimab, sintilimab, camrelizumab, or durvalumab	Postbronchodilator FEV1/FVC ratio <0.7	30	18	PFS	Age, sex, smoking, ECOG PS, tumor histology, tumor stage, and neoadjuvant cycles
Stevens, 2024	Australia	RC	152	67	63.2	III	Durvalumab	Clinically diagnosed COPD	50	26.1	PFS and OS	Age, sex, smoking, ECOG PS, CCI, CVD, tumor histology, stage, PD-L1 expression, and previous anticancer treatments
Chang, 2024	China	RC	57	60.6	94.7	I–IIIB	NR	Postbronchodilator FEV1/FVC ratio <0.7	18	18	PFS and OS	None
Hirakawa, 2025	Japan	RC	68	73	69.8	III–IV	NR	Clinically diagnosed COPD	31	11	PFS	Age, sex, smoking, BMI, PS, histology type, lines of treatment, and PD-L1 expression
Chan, 2025	Canada	RC	4306	64.6	51.1	I–IV	Atezolizumab, nivolumab, ipilimumab, or pembrolizumab	Clinically diagnosed COPD	2326	30	OS	Age, sex, ethnicity, tumor stage, SES, and previous anticancer treatments

ICI: Immune checkpoint inhibitor; RC: Retrospective cohort; FEV1: Forced expiratory volume in one second; FVC: Forced vital capacity; PFS: Progression-free survival; OS: Overall survival; ECOG PS: Eastern Cooperative Oncology Group performance status; CT: Computed tomography; PD-L1: Programmed death-ligand 1; NR: Not reported; CCI: Charlson Comorbidity Index; CVD: Cardiovascular disease; SES: Socioeconomic status; COPD: Chronic obstructive pulmonary disease.

## Results

### Study identification

The study selection process is summarized in Figure 1. A total of 569 potentially relevant records were initially identified through searches of three databases and citation screening of related articles. After removing 159 duplicates, 410 records remained. Screening of titles and abstracts led to the exclusion of 386 articles that did not meet the objectives of the meta-analysis. The full texts of the remaining 24 articles were independently reviewed by two authors, resulting in the exclusion of 11 studies for various reasons detailed in Figure 1. Ultimately, 13 studies were included in the quantitative analysis [20–32].

### Overview of the study characteristics

Table 1 summarizes the characteristics of the studies included in the meta-analysis. In total, 13 retrospective cohort studies—conducted between 2018 and 2025 in France, the United States, Korea, Japan, China, Australia, and Canada—were included [20–32]. These studies collectively involved 5564 patients with NSCLC. The mean age of patients ranged from 60.6 to 73.0 years, and the proportion of men ranged from 35.7% to 95.1%. Regarding cancer stage, 10 studies included patients with advanced NSCLC (stages III–IV) [20–26, 29, 30, 32], while the remaining three studies included patients with stages I–III [27, 31] or stages I–IV [28]. All patients received ICIs. COPD was diagnosed using different methods: five studies used a forced expiratory volume in 1 s to forced vital capacity ratio (FEV_1_/FVC) <0.7 [20, 22, 27, 30, 31]; five relied on clinical diagnosis recorded in medical charts [21, 26, 28, 29, 32]; and three diagnosed COPD based on CT-detected pulmonary emphysema [23–25]. At the time of ICI initiation, 2945 patients (52.9%) had COPD. The median follow-up duration across studies ranged from 10 to 42 months. PFS was reported in 11 studies [20–22, 24–27, 29–32], and OS in 10 studies [20–26, 28, 31, 32]. As shown in Table 1, survival outcomes were derived from either univariate or multivariate Cox proportional hazards regression analyses. The most adequately adjusted HRs and corresponding 95% CIs were extracted for data synthesis. Univariate analyses were conducted in five studies [20, 23, 26, 30, 31], while the remaining eight studies used multivariate analyses adjusted for factors, such as age, sex, tumor stage, and histology [21, 22, 24, 25, 27–29, 32]. The methodological quality of the included studies was generally moderate to high, with NOS scores ranging from five to nine (Table 2).

### Association between COPD and PFS

**Figure 2.**
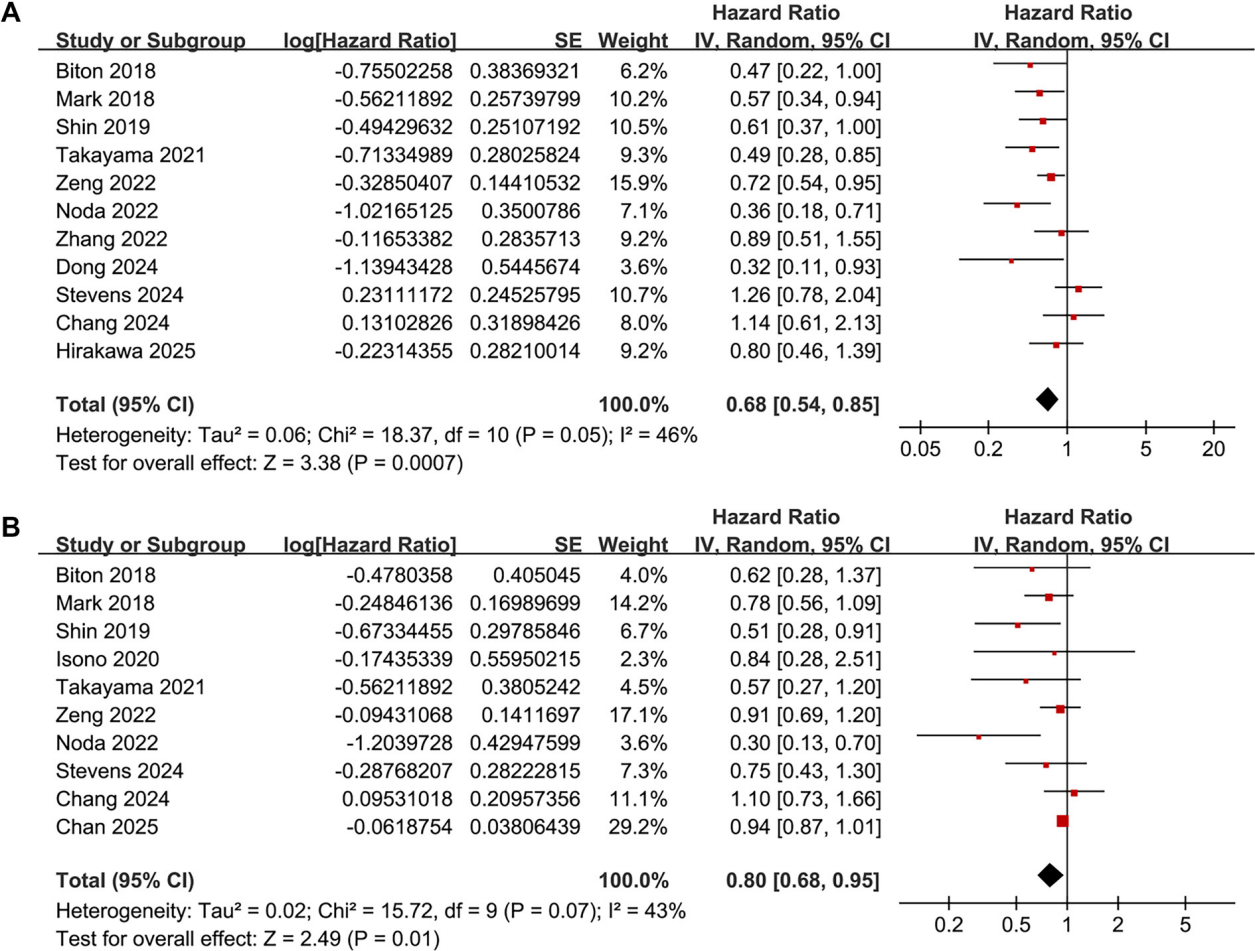
**Forest plots for the meta-analyses of the association between COPD and survival outcomes of patients with NSCLC on ICIs.** (A) Forest plots for the meta-analysis of PFS; (B) Forest plots for the meta-analysis of OS. NSCLC: Non-small cell lung cancer; PFS: Progression-free survival; OS: Overall survival; CI: Confidence interval; COPD: Chronic obstructive pulmonary disease; ICI: Immune checkpoint inhibitor.

Overall, 11 studies [20–22, 24–27, 29–32] reported on the association between COPD and PFS in patients with NSCLC receiving ICIs. Moderate heterogeneity was observed among these studies (Cochrane *Q* test *P* ═ 0.05; *I*^2^ ═ 46%). The pooled results indicated that COPD was associated with improved PFS in these patients (HR: 0.68, 95% CI: 0.54–0.85, *P* < 0.001; Figure 2A). Sensitivity analyses, conducted by excluding one study at a time, yielded consistent results (HR: 0.64–0.72, all <0.05). Furthermore, subgroup analyses showed that the association between COPD and PFS in NSCLC patients receiving ICIs was not significantly influenced by study country, patients’ mean age, proportion of male participants, COPD diagnostic method, follow-up duration, analytic model, or NOS scores (all *P* values for subgroup differences >0.05; Table 3).

**Table 2:** Study quality evaluation via the Newcastle-Ottawa Scale

**Study**	**Representativeness of the exposed cohort**	**Selection of the non-exposed cohort**	**Ascertainment of exposure**	**Outcome not present at baseline**	**Control for age and sex**	**Control for other confounding factors**	**Assessment of outcome**	**Enough long follow-up duration**	**Adequacy of follow-up of cohorts**	**Total**
Biton, 2018	0	1	1	1	0	0	1	1	1	6
Mark, 2018	0	1	1	1	1	1	1	1	1	8
Shin, 2019	0	1	1	1	1	1	1	1	1	8
Isono, 2020	0	1	1	1	0	0	1	0	1	5
Takayama, 2021	0	1	1	1	1	1	1	1	1	8
Zeng, 2022	1	1	1	1	0	0	1	1	1	7
Noda, 2022	0	1	1	1	1	1	1	1	1	8
Zhang, 2022	0	1	1	1	0	0	1	1	1	6
Dong, 2024	1	1	1	1	1	1	1	1	1	9
Stevens, 2024	0	1	1	1	1	1	1	1	1	8
Chang, 2024	0	1	1	1	0	0	1	1	1	6
Hirakawa, 2025	0	1	1	1	1	1	1	0	1	7
Chan, 2025	0	1	1	1	1	1	1	1	1	8

**Table 3:** Results of subgroup analysis

	**PFS**						**OS**				
**Variables**	**No. of studies**	**HR (95% CI)**	***I* ^2^**	***P* for subgroup effects**	***P* for subgroup difference**		**No. of studies**	**HR (95% CI)**	***I* ^2^**	***P* for subgroup effects**	***P* for subgroup difference**
*Countries*											
Asian countries	8	0.66 [0.52, 0.84]	35%	<0.001			6	0.71 [0.50, 1.00]	56%	0.05	
Western countries	3	0.72 [0.39, 1.34]	72%	0.30	0.80		4	0.93 [0.86, 0.99]	0%	0.03	0.14
*Mean ages*											
<65 years	4	0.74 [0.49, 1.12]	42%	0.16			3	0.88 [0.65, 1.19]	56%	0.40	
≥65 years	7	0.65 [0.49, 0.86]	53%	0.003	0.62		7	0.75 [0.60, 0.92]	16%	0.007	0.39
*Men*											
<75%	5	0.66 [0.42, 1.01]	65%	0.06			5	0.75 [0.57, 0.99]	58%	0.05	
≥75%	6	0.69 [0.54, 0.89]	29%	0.004	0.83		5	0.82 [0.62, 1.08]	31%	0.16	0.66
*Diagnosis of COPD*											
FEV1/FVC ratio < 0.7	5	0.69 [0.47, 1.00]	38%	0.05			3	0.74 [0.44, 1.25]	60%	0.26	
Clinically diagnosed COPD	4	0.79 [0.59, 1.07]	47%	0.13			4	0.93 [0.86, 0.99]	0%	0.03	
CT diagnosed PE	2	0.43 [0.28, 0.67]	0%	<0.001	0.08		3	0.50 [0.29, 0.86]	16%	0.01	0.06
*Mean follow-up duration*											
<20 months	4	0.72 [0.48, 1.08]	38%	0.11			3	0.79 [0.46, 1.37]	55%	0.41	
≥20 months	7	0.66 [0.49, 0.88]	55%	0.005	0.74		7	0.80 [0.66, 0.97]	46%	0.02	0.98
*Analytic models*											
Univariate	4	0.77 [0.59, 1.01]	17%	0.06			4	0.93 [0.75, 1.15]	0%	0.51	
Multivariate	7	0.62 [0.44, 0.86]	56%	0.004	0.30		6	0.69 [0.52, 0.92]	64%	0.01	0.11
*NOS*											
<7	3	0.82 [0.51, 1.30]	38%	0.40			3	0.96 [0.68, 1.36]	0%	0.82	
≥7	8	0.64 [0.49, 0.83]	50%	<0.001	0.38		7	0.76 [0.62, 0.94]	57%	0.01	0.26

PFS: Progression-free survival; OS: Overall survival; HR: Hazard ratio; CI: Confidence interval; *I*^2^: Heterogeneity index; FEV1: Forced expiratory volume in one second; FVC: Forced vital capacity; COPD: Chronic obstructive pulmonary disease; CT: Computed tomography; PE: Pulmonary emphysema; NOS: Newcastle-Ottawa Scale.

### Association between COPD and OS

The pooled results of 10 studies [20–26, 28, 31, 32] indicated that COPD was associated with improved OS in patients with NSCLC receiving ICIs (HR: 0.80; 95% CI: 0.68–0.95; *P* ═ 0.01; Figure 2B), with moderate heterogeneity (Cochrane *Q* test *P* ═ 0.07; *I*^2^ ═ 43%). Sensitivity analysis, performed by excluding one study at a time, did not significantly alter the results (HR range: 0.75–0.89, *P* all <0.05). Similarly, subgroup analyses based on study country, mean age, proportion of male participants, COPD diagnosis method, follow-up duration, analytic model, or NOS scores did not significantly affect the association (all subgroup *P* values >0.05; Table 3). Furthermore, univariate meta-regression analysis showed no significant modification of results based on sample size, mean age, proportion of men, follow-up duration, or study quality scores (*P* all >0.05; Table 4).

**Table 4:** Results of univariate meta-regression analysis

**Variables**	**HR for PFS**			**HR for OS**		
	**Coefficient**	**95% CI**	***P* values**	**Coefficient**	**95% CI**	***P* values**
Sample size	0.0022	−0.0053 to 0.0096	0.53	0.000054	−0.000066 to 0.000175	0.33
Mean age (years)	−0.030	−0.116 to 0.055	0.44	−0.072	−0.159 to 0.015	0.16
Men (%)	0.0086	−0.0064 to 0.0237	0.23	0.0067	−0.0066 to 0.0199	0.28
Follow-up duration (months)	0.014	−0.021 to 0.048	0.40	0.017	−0.017 to 0.051	0.28
NOS	−0.18	−0.48 to 0.12	0.21	−0.11	−0.38 to 0.15	0.35

HR: Hazard ratio; PFS: Progression-free survival; OS: Overall survival; CI: Confidence interval; NOS: Newcastle–Ottawa Scale.

### Publication bias

**Figure 3.**
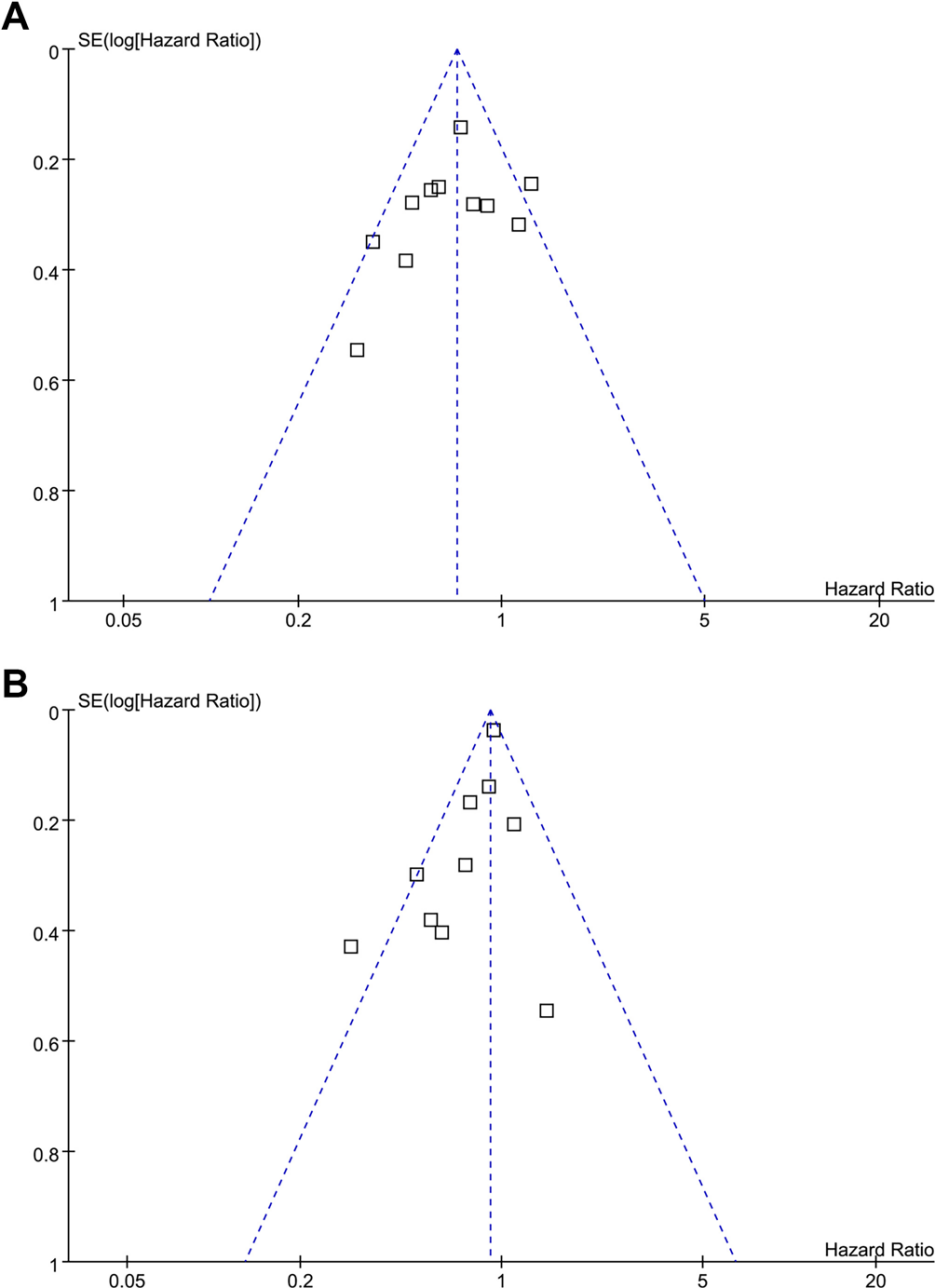
**Funnel plots for evaluating the publication bias underlying the meta-analyses**. (A) Funnel plots for the meta-analysis of the association between COPD and PFS of patients with NSCLC receiving ICIs and (B) Funnel plots for the meta-analysis of the association between COPD and OS of patients with NSCLC receiving ICIs. NSCLC: Non-small cell lung cancer; PFS: Progression-free survival; OS: Overall survival; COPD: Chronic obstructive pulmonary disease; ICI: Immune checkpoint inhibitor.

The funnel plots for the meta-analyses assessing the associations between COPD and PFS/OS in patients with NSCLC receiving ICIs are presented in Figure 3A and 3B. Visual inspection of the plots suggests symmetry, indicating a low risk of publication bias. These observations are further supported by Egger’s regression analyses (PFS: *P* ═ 0.47; OS: *P* ═ 0.42).

## Discussion

Our meta-analysis provides comprehensive evidence on the influence of COPD on the survival of patients with NSCLC treated with ICIs. The pooled results from 13 retrospective cohort studies, including 5564 patients, indicate that COPD is associated with improved PFS and OS in patients receiving ICIs. These findings challenge the conventional notion that COPD universally worsens lung cancer outcomes and suggest that COPD may confer a survival advantage in NSCLC patients undergoing ICI therapy. The potential mechanisms underlying the observed association between COPD and improved ICI efficacy remain an area of active investigation [17, 19]. Several studies have proposed that COPD-related immune dysregulation may enhance the therapeutic effects of ICIs [17]. Patients with COPD exhibit chronic inflammation and immune alterations that may increase tumor immunogenicity [39]. Notably, COPD is associated with increased infiltration of CD8+ tumor-infiltrating lymphocytes (TILs), which play a crucial role in anti-tumor immunity [40]. A study by Biton et al. demonstrated that COPD patients with NSCLC exhibited higher levels of PD-1/TIM-3 coexpression in CD8+ T cells, suggesting increased T-cell exhaustion and a potential for greater responsiveness to PD-1 blockade [20]. In addition, Th1 cell populations were observed to be expanded in both lung tissue and tumor microenvironments in patients with COPD and NSCLC, which may also be associated with better responsiveness to ICIs [21]. Furthermore, PD-L1 expression—a key biomarker for ICI efficacy—appears to be upregulated in COPD-associated NSCLC, which may partly explain the improved response to ICIs observed in our meta-analysis [41, 42]. Increased TMB has also been reported in COPD-associated lung cancer [41], which is another well-recognized predictor of enhanced ICI efficacy. Collectively, these findings suggest that the inflammatory milieu in COPD may foster an immune microenvironment more conducive to ICI therapy. Consistently, a recent study [43] also found better PFS with chemotherapy in NSCLC patients with COPD, possibly due to reduced systemic inflammation and enhanced immune activation. These mechanisms may similarly contribute to the improved ICI efficacy observed in patients with NSCLC and COPD. Subgroup analyses in our study revealed that the association between COPD and improved survival was consistent across various study characteristics, including geographic region, patient age, sex distribution, COPD diagnostic criteria (spirometry, clinical diagnosis, or CT-diagnosed emphysema), and follow-up duration. Notably, the survival benefit was more pronounced in studies that reported multivariate-adjusted HRs, suggesting that confounding factors, such as smoking history, tumor histology, and comorbidities did not fully account for the observed association. This supports the hypothesis that COPD itself—rather than associated lifestyle factors—contributes to enhanced ICI efficacy. Moreover, results of the univariate meta-regression analysis did not indicate that characteristics, such as sample size, mean age, proportion of men, follow-up duration, or NOS scores significantly modified the outcomes, further validating the robustness of our findings. Beyond survival outcomes, the interaction between ICIs and COPD itself is of particular interest. Recent evidence suggests that PD-1 blockade may modulate lung inflammation and improve pulmonary function in COPD patients [44, 45]. A prospective study by Suzuki et al. demonstrated that COPD patients treated with nivolumab experienced significant increases in FVC and FEV1, suggesting a potential beneficial effect of PD-1 inhibition on lung function [44]. Furthermore, although fractional exhaled nitric oxide (FeNO) levels were significantly elevated after ICI therapy—indicating increased airway inflammation—there was no significant worsening of dyspnea or COPD exacerbations [44]. Notably, the study also reported a numerically higher tumor response rate in COPD patients, reinforcing the hypothesis that immune changes associated with COPD may enhance ICI efficacy [44]. However, while this study suggested that ICIs may improve NSCLC survival without worsening COPD symptoms, a more recent study reported an increased risk of COPD exacerbations following ICI therapy [45]. In a large retrospective cohort from the United States, COPD patients treated with ICIs experienced higher rates of exacerbations and respiratory-related hospitalizations, likely due to immune-related inflammation [45]. The discrepancy between these findings highlights the complexity of ICI effects on COPD and underscores the need for further research to optimize patient management.

Clinically, our findings suggest that COPD should not be considered a contraindication to ICI therapy in NSCLC. On the contrary, the presence of COPD may serve as a potential biomarker for enhanced ICI efficacy. However, due to the risk of COPD exacerbation following ICI treatment, close monitoring and proactive symptom management are essential to ensure optimal patient outcomes. Future research should prioritize prospective studies to validate our findings and clarify the mechanisms by which COPD influences the tumor immune microenvironment. Additionally, investigating the impact of COPD therapies—such as inhaled corticosteroids and bronchodilators—on ICI efficacy is warranted, as these treatments may modulate immune responses. Our meta-analysis has several strengths. It provides the most up-to-date and comprehensive synthesis of evidence on this topic, incorporating an extensive literature search across multiple databases. The inclusion of cohort studies with longitudinal follow-up enhances the reliability of our findings, as these studies offer robust estimates of real-world survival outcomes. Furthermore, we conducted multiple sensitivity, subgroup, and meta-regression analyses to assess the robustness of our results, minimizing potential bias and confounding. Nonetheless, our study has limitations. First, all included studies were retrospective, which inherently introduces risks of recall and selection bias. Second, the potential impact of COPD-specific treatments, such as inhaled corticosteroids and bronchodilators, on survival outcomes could not be evaluated due to the lack of detailed treatment data in most studies. These medications may influence systemic inflammation or immune responses and could have confounded the observed associations. Third, due to the observational design of the included studies, causality cannot be established, and residual confounding remains a concern. Moreover, we were unable to assess the influence of certain patient and study characteristics—such as concurrent medications, tumor histology, and the presence of immune-related adverse events—because individual patient data were not available. These factors may have affected the observed relationship between COPD and ICI response. Some included studies also enrolled patients with early-stage NSCLC (stages I–II) receiving ICIs in the neoadjuvant or adjuvant setting, where survival outcomes are more likely to be influenced by surgical and perioperative factors than by systemic therapy. Although most studies included patients with unresectable stages III–IV disease, subgroup analyses by cancer stage could not be performed due to the lack of individual-level data, which should be considered when interpreting the pooled results. Importantly, the impact of ICI therapy on COPD progression was not evaluated in the included studies, despite emerging evidence suggesting that COPD symptoms may worsen after treatment. Future work should explore strategies to mitigate ICI-associated COPD exacerbations while preserving therapeutic efficacy. Additionally, not all studies used spirometry to define COPD; some relied on clinical diagnoses or CT evidence of emphysema, which may not meet standardized diagnostic criteria. This heterogeneity in exposure definitions may introduce misclassification bias and affect interpretation of the pooled findings. Finally, the interpretation of funnel plots and Egger’s test should be made cautiously, given the limited number of included studies. A nonsignificant Egger’s test does not exclude the possibility of publication bias under such conditions.

## Conclusion

In conclusion, this meta-analysis demonstrates that COPD is associated with improved survival in NSCLC patients treated with ICIs. Potential underlying mechanisms include enhanced tumor immunogenicity, increased PD-L1 expression, and higher TMB in COPD-associated lung cancer. However, recent evidence suggests that ICI therapy may worsen COPD symptoms, highlighting the need for careful patient monitoring. These findings offer new insights into the interaction between COPD and immunotherapy response, underscoring the need for further prospective validation and mechanistic studies.

## Supplemental data

**PubMed**

(((“Pulmonary Disease, Chronic Obstructive” [Mesh]) OR “chronic obstructive pulmonary disease” OR “COPD” OR “chronic obstructive lung disease” OR “chronic obstructive airway disease” OR “emphysema” OR “chronic airflow limitation” OR “chronic airway obstruction”)) AND (((“Carcinoma, Non-Small-Cell Lung” [Mesh]) OR “Non-Small Cell Lung Cancer” OR “Non-Small Cell Carcinoma” OR “NSCLC” OR “Lung Adenocarcinoma” OR “Lung Squamous Cell Carcinoma” OR “Lung Neoplasms” OR “Lung Cancer”)) AND (((“Immune Checkpoint Inhibitors” [Mesh]) OR “Immune Checkpoint Inhibitors” OR “ICI” OR “Programmed Cell Death Protein 1” OR “PD-1” OR “PD-L1” OR “Cytotoxic T-Lymphocyte-Associated Protein 4” OR “CTLA-4” OR “Pembrolizumab” OR “Nivolumab” OR “Atezolizumab” OR “Durvalumab” OR “Cemiplimab” OR “Avelumab” OR “Ipilimumab” OR “Tremelimumab”))

**Embase**

(“chronic obstructive pulmonary disease”/exp OR “chronic obstructive pulmonary disease” OR “COPD” OR “chronic obstructive lung disease” OR “chronic obstructive airway disease” OR “emphysema” OR “chronic airflow limitation” OR “chronic airway obstruction”) AND (“non small cell lung cancer”/exp OR “non small cell lung cancer” OR “non small cell carcinoma” OR “NSCLC” OR “lung adenocarcinoma” OR “lung squamous cell carcinoma” OR “lung neoplasms” OR “lung cancer”) AND (“immune checkpoint inhibitor”/exp OR “immune checkpoint inhibitors” OR “ICI” OR “programmed cell death protein 1” OR “PD-1” OR “PD-L1” OR “cytotoxic t lymphocyte associated protein 4” OR “CTLA-4” OR “pembrolizumab” OR “nivolumab” OR “atezolizumab” OR “durvalumab” OR “cemiplimab” OR “avelumab” OR “ipilimumab” OR “tremelimumab”)

**Web of Science**

TS ═ (“chronic obstructive pulmonary disease” OR “COPD” OR “chronic obstructive lung disease” OR “chronic obstructive airway disease” OR “emphysema” OR “chronic airflow limitation” OR “chronic airway obstruction”) AND TS ═ (“Non-Small Cell Lung Cancer” OR “Non-Small Cell Carcinoma” OR “NSCLC” OR “Lung Adenocarcinoma” OR “Lung Squamous Cell Carcinoma” OR “Lung Neoplasms” OR “Lung Cancer”) AND TS ═ (“Immune Checkpoint Inhibitors” OR “ICI” OR “Programmed Cell Death Protein 1” OR “PD-1” OR “PD-L1” OR “Cytotoxic T-Lymphocyte-Associated Protein 4” OR “CTLA-4” OR “Pembrolizumab” OR “Nivolumab” OR “Atezolizumab” OR “Durvalumab” OR “Cemiplimab” OR “Avelumab” OR “Ipilimumab” OR “Tremelimumab”)

## Data Availability

The data that support the findings of this study are available from the corresponding author upon reasonable request.
